# Whole-Genome Analysis of a Novel Fish Reovirus (MsReV) Discloses Aquareovirus Genomic Structure Relationship with Host in Saline Environments

**DOI:** 10.3390/v7082820

**Published:** 2015-08-03

**Authors:** Zhong-Yuan Chen, Xiao-Chan Gao, Qi-Ya Zhang

**Affiliations:** State Key Laboratory of Freshwater Ecology and Biotechnology, Institute of Hydrobiology, Chinese Academy of Sciences, Wuhan 430072, China; E-Mails: chenzy@ihb.ac.cn (Z.-Y.C.); gaoxiaochan@ihb.ac.cn (X.-C.G.)

**Keywords:** aquareovirus genome, largemouth bass *micropterus salmoides* reovirus (MsReV), fusion-associated small transmembrane (FAST) protein, host environments

## Abstract

Aquareoviruses are serious pathogens of aquatic animals. Here, genome characterization and functional gene analysis of a novel aquareovirus, largemouth bass *Micropterus salmoides* reovirus (MsReV), was described. It comprises 11 dsRNA segments (S1–S11) covering 24,024 bp, and encodes 12 putative proteins including the inclusion forming-related protein NS87 and the fusion-associated small transmembrane (FAST) protein NS22. The function of NS22 was confirmed by expression in fish cells. Subsequently, MsReV was compared with two representative aquareoviruses, saltwater fish turbot *Scophthalmus maximus* reovirus (SMReV) and freshwater fish grass carp reovirus strain 109 (GCReV-109). MsReV NS87 and NS22 genes have the same structure and function with those of SMReV, whereas GCReV-109 is either missing the coiled-coil region in NS79 or the gene-encoding NS22. Significant similarities are also revealed among equivalent genome segments between MsReV and SMReV, but a difference is found between MsReV and GCReV-109. Furthermore, phylogenetic analysis showed that 13 aquareoviruses could be divided into freshwater and saline environments subgroups, and MsReV was closely related to SMReV in saline environments. Consequently, these viruses from hosts in saline environments have more genomic structural similarities than the viruses from hosts in freshwater. This is the first study of the relationships between aquareovirus genomic structure and their host environments.

## 1. Introduction

Reoviruses are non-enveloped, icosahedral, double-stranded RNA (dsRNA) viruses which have double-layered capsids and genomes consisting of 9 to 12 genome segments [1]. Genus *Aquareovirus* belonging to the family *Reoviridae* can infect a wide variety of aquatic animals, including crustacean, shellfish, and fish [2]. These viruses are capable of causing severe hemorrhagic disease in fish and syncytia in cell culture, and have been isolated from hosts in both freshwater and saline environments worldwide [3,4,5,6,7,8,9,10,11,12]. Viruses in genus *Aquareovirus* have 11 dsRNA segments (S1–S11) [2]. Recently, a fish reovirus (piscine reovirus, PRV), which is proposed as a tentative new member of the family *Reoviridae*, has been identified to have 10 dsRNA segments [13]. Most segments of aquareovirus have only one open reading frame (ORF), and the genome usually encodes seven major structural proteins (VP1–VP7) and five major nonstructural proteins.

Phylogenetic analysis has been an increasingly efficient tool to examine aquatic animal virus epidemiology, and to infer common ancestors and their host relationships [14,15,16,17,18]. Comparative sequence analysis shows interesting similarities and dissimilarities among some equivalent genome segments of the reoviruses [19,20]. A similar genomic structure occurs in a closely related species [21,22]. Initial attempts to examine genetic variation among aquareovirus isolates primarily used partial or full-length genome sequences [23]. The primary goal was to distinguish between different aquareovirus strains. In recent years, aquareoviruses were recognized with high genomic variability [24,25]. The expanding knowledge of aquareovirus genetics has led to a number of groups proposing sequence-based typing schemes. For example, the phylogenetic tree of RNA dependent RNA polymerase (RdRp) revealed evidence for genetic relatedness and genomic diversity of aquareoviruses [26,27]. The phylogenetic comparison of reovirus genetic segments allowed the identification of reoviruses into *Spinareovirinae* and *Sedoreovirinae* subfamilies [1,28].

Until now, more than 15 aquareovirus genomes have been completely sequenced [12], including turbot *Scophthalmus maximus* reovirus (SMReV), and grass carp reovirus strain 109 (GCReV-109), as previously reported by our laboratory [24,26], and largemouth bass *Micropterus salmoides* reovirus (MsReV) described in this article. They have been found by way of the molecular variation that occurs throughout reoviral genomes, which are correlated with geographical distribution, classification (e.g., *Aquareovirus* and *Orthoreovirus*), morphological features (e.g., turreted reoviruses *Spinareovirinae* and non-turreted reoviruses *Sedoreovirinae*) [16,29,30]. Genomic variability may also assist in determining viruses with distinct cytopathic and pathogenic properties (e.g., causing syncytia and inclusion body formation) [26,31]. The properties of viral genome such as its size and chemical composition are identified as major determinants of evolutionary rates [32]. Some putative genes and the probable functions of encoded proteins in aquareoviruses have been reported [6,25,27,33,34,35]. Aquareovirus GCRV S4 encoding NS80 ensures its self-aggregation to form viral factories like structures (VFLS) and recruitment of viral proteins [31]. Aquareoviruses have been used as a model to understand the structural basis and pathogenesis of reovirus [26,31,36]. An unexpected challenge that has arisen from aquareoviruses is the viral genetic diversity, and the prevention and control of aquareoviruses remains largely unaddressed as they are known to be very limited in the viral pathogen–host fish. Although genome diversity has been reported among aquareoviruses [12,24,25,26], there is finite information on the natural processes that contribute to genome diversity, and it is still unclear whether the genome structure of aquareovirus is reflective of the interrelationships between the virus and host environment.

To assess the genomic variability of aquareoviruses and the relationships between the viruses and their host environments, here we investigated the aquareovirus genomic structure relationship with hosts in saline environments based on new as well as previously published sequence information, with a comparison of equivalent genomic segments and phylogenetic analysis. Moreover, the phenotype associated with the pathogenicity of specific gene MsReV *NS22* was tested by construction and expression of plasmid with deletions or mutations.

## 2. Materials and Methods

### 2.1. Virus Isolation, Electron Microscopy and Electrophoretic Analysis

Diseased largemouth bass were collected in Hubei province of China in May 2010. Liver, spleen and kidney tissues were sampled and homogenized as previously described [26]. The suspension was centrifuged at 2000 *g* for 30 min and then filtered through a sterile 0.45 µm filter (Millipore, Billerica, MA, USA). The filtered supernatant was inoculated into confluent monolayers of bluegill fry (BF-2), chinook salmon embryo (CHSE-214), epithelioma papulosum cyprini (EPC), fathead minnow (FHM), grass carp fins (GCF) and grass carp ovary (GCO) cell lines in TC199 medium containing 5% fetal bovine serum at 15 °C, 20 °C or 25 °C. Inoculated cell cultures were checked daily for cytopathic effects. The original viral isolate was adapted to cell culture through at least three passages on these cell lines. The optimal temperature for virus propagation was assayed by infection of GCF cell monolayers at 15 °C, 20 °C or 25 °C. Viral titers were measured on the basis of 50% tissue culture infective dose (TCID_50_) mL^−1^ as described previously [10].

Virus particles were purified from cell culture-amplified virus stocks as described previously [26]. Purified virus particles were negatively stained with 2% (*w/v*) phosphotungstic acid, and then examined with the Hitachi HT-7700 electron microscope (Hitachi, Tokyo, Japan).

Virus dsRNA was extracted from purified virus particles using Trizol Reagent according to the manufacture’s instruction (Invitrogen, Carlsbad, CA, USA). The extracted dsRNA was analyzed on a 15% polyacrylamide gel in 0.5× Tris-borate/EDTA (TBE) buffer, and then visualized by sliver staining. Genomic dsRNA from *Scophthalmus maximus* reovirus (SMReV) and grass carp reovirus strain 109 (GCReV-109) maintained in our laboratory [24,26] were prepared and used as molecular mass size markers.

### 2.2. Viral Genome Sequencing

The cDNA from virus dsRNA was synthesized using the single-primer amplification technique [26]. Briefly, an oligodeoxyribonucleotide primer (TC1: 5′ PO_4_-CCCGCCATCCTCACTTAGACT-NH_2_ 3′) was ligated to both of the 3′ ends of the dsRNA segments by T4 RNA ligase (TaKaRa, Dalian, China). After the reaction, dsRNA was denatured at 94 °C for 5 min in the presence of 15% dimethyl sulfoxide (DMSO) and then cooled rapidly on ice. RNA was then removed by adding NaOH and the cDNA was annealed at 65 °C overnight. The first strand cDNA of the genome segments were synthesized using M-MLV (Promega, Madison, WI, USA), and then purified by a Sephacryl S-400 spin column (Promega). The amplification of the cDNA was performed using the complementary primer (TC2: 5′AGTCTAAGTGAGGATGGCGGG 3′). PCR products were electrophoresed on 1% agarose gels, and all visible bands were purified and ligated into the pMD18-T vector (TaKaRa). The positive clones were sequenced on an ABI 3730XL DNA analyzer (Sangon, Shanghai, China).

### 2.3. Sequence Analysis and Comparison

The nucleotide sequences and deduced amino acid sequences were analyzed using the EditSeq program (DNASTAR 5.0). Homology searches of nucleic acid and protein databases were performed using BLAST at the National Centre for Biotechnology Information server. Multiple sequence alignments were performed using Clustal X 1.83 program, and sequence identities were calculated using the Clusta W method in the MegAlign program (DNASTAR 5.0). Transmembrane helices were predicted using TMHMM 2.0 [37]. The coiled regions in MsReV NS87 protein were predicted using the COILS Server (http://embnet.vital-it.ch/software/COILS_form.html). The equivalent genome segments and proteins between MsReV and two other representative aquareoviruses, SMReV and GCReV-109, were analyzed and shown in a schematic diagram.

### 2.4. Plasmid Construction, Transfection and Cell Staining

To analyze the function of MsReV NS22 protein, DNA fragments that contained different regions of S7, including full-length NS22-coding gene (1–613), deletions (14–613, 15–613, 17–613) and point mutations (1-613/∆14, 1-613/∆18), were amplified from cDNAs obtained above using primers designed by methods previously described [26]. The PCR products were cut and ligated into pcDNA3.1(+) vector (Invitrogen) with corresponding enzymes. A recombinant eukaryotic vector, pEGFP-NS22, was also constructed by cloning the entire NS22 gene into pEGFP-N3 vector (Clontech, Palo Alto, CA, USA). All constructed plasmids were confirmed by restriction digestion and DNA sequencing.

GCF cells grown in 24-well plates were transfected with plasmids using Lipofectamine 2000 (Invitrogen) according to the manufacturer’s instructions. At 48 h post transfection, the cells were fixed with methanol, stained with Wright-Giemsa staining, and examined with light microscope (Leica, Wetzlar, Germany). Alternatively, cells transfected with the plasmid pEGFP-NS22 were fixed with 4% paraformaldehyde at 48 h post transfection. The fixed cells were stained with Hoechst 33342 and observed by fluorescence microscopy as described previously [38,39].

### 2.5. Phylogenetic Analysis

Phylogenetic analysis was performed based on the alignment of the concatenated sequences of seven structural proteins that are conserved in all sequenced aquareoviruses (Table 1). The seven structural proteins from 12 other aquareoviruses were rearranged as continuous amino acid sequences with the same order as MsReV. The concatenated protein sequences were then aligned with the Clustal X 1.83 program, and phylogenetic tree was constructed using the neighbor-joining method with 1000 bootstrap replicates in MEGA5 software [40]. GenBank accession numbers of the aquareovirus sequences used for analysis were shown in Table 1.

**Table 1:** Summary of genome segments and encoded structural proteins of 13 aquareoviruses and percent sequence identities of the concatenated seven structural proteins between MsReV and other aquareoviruses.

Different Aquareoviruses	Genome Segment/Length (bp)	Coding Segment	S1		S2		S3		S5		S6		S8		S10		Coding Segment/GenBank acc. No.	Identity (%) of the Concatenated Seven Structural Proteins between MsReV and Other Aquareoviruses
		Structural Protein	VP1		VP2		VP3		VP4		VP5		VP6		VP7			
			Size (aa)	MW (kDa)	Size (aa)	MW (kDa)	Size (aa)	MW (kDa)	Size (aa)	MW (kDa)	Size (aa)	MW (kDa)	Size (aa)	MW (kDa)	Size (aa)	MW (kDa)		
MsReV	S1/3947 S2/3866 S3/3687 S4/2622 S5/2242 S6/2056 S7/1399 S8/1317 S9/1118 S10/987 S11/783		**1297**	140.91	**1274**	141.24	**1209**	131.05	**722**	79.82	**653**	69.04	**417**	45.39	**298**	32.38	S1/KJ740725 S2/KJ740726 S3/KJ740727 S5/KJ740729 S6/KJ740730 S8/KJ740732 S10/KJ740734	**100**
SMReV	S1/3947 S2/3866 S3/3687 S4/2640 S5/2241 S6/2057 S7/1399 S8/1317 S9/1118 S10/986 S11/784		**1297**	141.40	**1274**	140.97	**1209**	131.10	**730**	80.52	**653**	69.25	**417**	45.18	**298**	32.18	S1/HM989930 S2/HM989931 S3/HM989932 S5/HM989934 S6/HM989935 S8/HM989937 S10/HM989939	**91.2**
CHSRV	S1/3947 S2/3867 S3/3690 S4/partial S5/2242 S6/2052 S7/1395 S8/1317 S9/1118 S10/985 S11/783		**1297**	140.93	**1240**	137.58	**1210**	131.95	**723**	80.15	**643**	68.89	**417**	45.34	**298**	32.41	S1/AF418294 S2/AF418295 S3/AF418296 S5/AF418298 S6/AF418299 S8/AF418301 S10/AF418303	**85.5**
									**S5**		**S6**							
									**VP5**		**VP4**							
									**Size (aa)**	**MW (kDa)**	**Size (aa)**	**MW (kDa)**						
GCRV-873	S1/3949 S2/3877 S3/3702 S4/2320 S5/2239 S6/2039 S7/1414 S8/1296 S9/1130 S10/909 S11/820		**1299**	141.41	**1274**	141.54	**1214**	132.10	**728**	80.24	**648**	68.60	**412**	44.58	**276**	29.81	S1/AF260511 S2/AF260512 S3/AF260513 S5/AF403391 S6/AF403392 S8/AF403394 S10/AF403396	**48.5**
GSRV	S1/3949 S2/3877 S3/3702 S4/2320 S5/2239 S6/2039 S7/1414 S8/1297 S9/1130 S10/909 S11/820		**1299**	141.27	**1274**	141.59	**1214**	132.06	**728**	80.25	**648**	68.56	**412**	44.59	**276**	29.79	S1/NC_005166 S2/NC_005167 S3/NC_005168 S5/NC_005170 S6/NC_005171 S8/NC_005173 S10/NC_005175	**48.4**
AGCRV	S1/3949 S2/3876 S3/3709 S4/2293 S5/2237 S6/2042 S7/1356 S8/1305 S9/1125 S10/912 S11/772		**1289**	141.11	**1274**	141.98	**1215**	131.93	**728**	80.14	**650**	68.99	**413**	44.97	**273**	30.36	S1/NC_010584 S2/NC_010585 S3/NC_010586 S5/NC_010588 S6/NC_010589 S8/NC_010591 S10/NC_010593	**47.8**
													**S9**		**S11**			
													**VP6**		**VP7**			
													**Size (aa)**	**MW (kDa)**	**Size (aa)**	**MW (kDa)**		
GCRV106	S1/3927 S2/3867 S3/3753 S4/2263 S5/2229 S6/2030 S7/1604 S8/1556 S9/1320 S10/1124 S11/1105		**1294**	143.68	**1273**	142.64	**1232**	135.82	**726**	80.61	**650**	68.37	**418**	48.00	**310**	35.46	S1/KC201166 S2/KC201167 S3/KC201168 S5/KC201170 S6/KC201171 S9/KC201174 S11/KC201176	**30.7**
GCReV-109	S1/3928 S2/3867 S3/3753 S4/2263 S5/2230 S6/2028 S7/1604 S8/1560 S9/1320 S10/1124 S11/1027		**1294**	143.72	**1273**	142.08	**1232**	135.73	**726**	80.64	**650**	68.21	**418**	47.96	**310**	35.48	S1/KF712475 S2/KF712476 S3/KF712477 S5/KF712479 S6/KF712480 S9/KF712483 S11/KF712485	**30.6**
GCRV918	S1/3927 S2/3867 S3/3753 S4/2263 S5/2229 S6/2030 S7/1604 S8/1556 S9/1320 S10/1124 S11/1107		**1294**	143.61	**1273**	142.64	**1232**	135.85	**726**	80.58	**650**	68.29	**418**	48.09	**310**	35.48	S1/KC201177 S2/KC201178 S3/KC201179 S5/KC201181 S6/KC201182 S9/KC201185 S11/KC201187	**30.6**
GCRV-GD108	S1/3928 S2/3867 S3/3752 S4/2263 S5/2230 S6/2028 S7/1604 S8/1560 S9/1320 S10/1124 S11/1027		**1294**	143.43	**1273**	142.61	**1232**	135.78	**726**	80.72	**650**	68.28	**418**	47.99	**310**	35.43	S1/HQ231198 S2/HQ231199 S3/HQ231200 S5/HQ231202 S6/HQ231208 S9/HQ231205 S11/HQ231207	**30.6**
GCRV-HuNan794	S1/3927 S2/3867 S3/3753 S4/2263 S5/2229 S6/2030 S7/1604 S8/1556 S9/1320 S10/1124 S11/1107		**1294**	143.68	**1273**	142.62	**1232**	135.66	**726**	80.65	**650**	68.37	**418**	48.03	**310**	35.43	S1/KC238676 S2/KC238677 S3/KC238678 S5/KC238680 S6/KC238681 S9/KC238684 S11/KC238686	**30.6**
GCRV-HeNan988	S1/3927 S2/3867 S3/3753 S4/2263 S5/2229 S6/2030 S7/1604 S8/1556 S9/1320 S10/1124 S11/1107		**1294**	143.72	**1273**	142.66	**1232**	135.45	**726**	80.62	**650**	68.37	**418**	48.10	**310**	35.48	S1/KC847320 S2/KC847321 S3/KC847322 S5/KC847324 S6/KC847325 S9/KC847328 S11/KC847330	**30.6**
GCRV-HZ08	S1/3927 S2/3870 S3/3753 S4/2263 S5/2229 S6/2030 S7/1604 S8/1560 S9/1320 S10/1124 S11/1027		**1294**	143.66	**1273**	143.10	**1232**	135.86	**726**	80.54	**650**	68.37	**418**	47.87	**310**	35.44	S1/GQ896334 2/GQ896335 S3/GU350742 S5/GQ896336 S6/GQ896337 S9/GU350746 S11/GU350748	**30.4**

bp, base pairs; aa, amino acids; MW, molecular weight; SMReV, *Scophthalmus maximus* reovirus; CHSRV, chum salmon reovirus; GCRV-873, grass carp reovirus 873; GSRV, golden shiner reovirus; AGCRV, American grass carp revirus; GCRV106, grass carp reovirus 106; GCReV-109, grass carp reovirus 109; GCRV918, grass carp reovirus 918, GCRV-GD108, grass carp reovirus GD108, GCRV-HuNan794, grass carp reovirus HuNan794, GCRV-HeNan988, grass carp reovirus HeNan988, GCRV-HZ08, grass carp reovirus HZ08.

## 3. Results

### 3.1. Identification of MsReV and Electrophoretic Pattern of Viral Genome Segments

**Figure 1:**
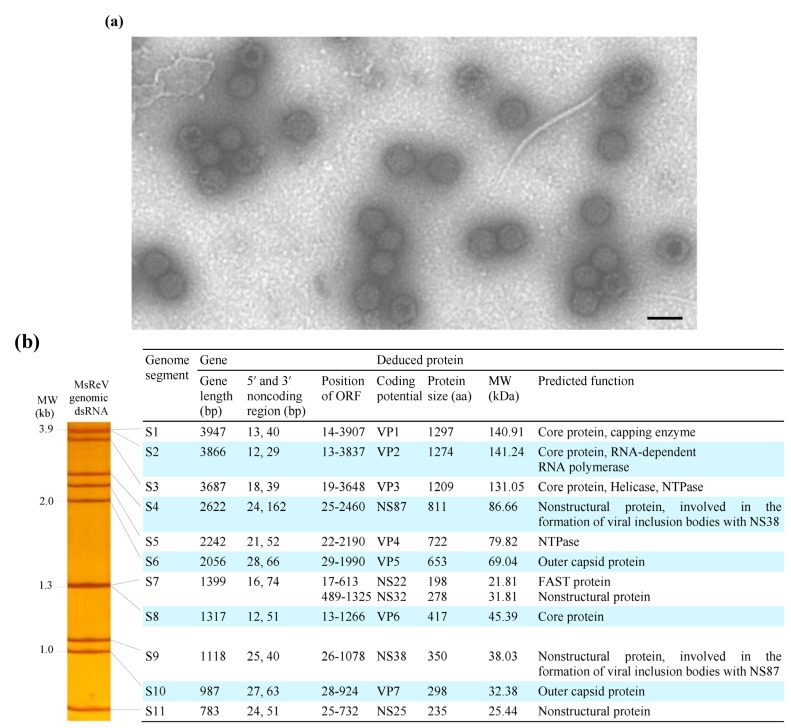
Morphology and genome organization of MsReV. (**a**) Electron micrograph of negatively stained MsReV particles. The virions are approximately 70–80 nm in diameter and have double-layered capsids. Bar = 100 nm; (**b**) Left panel, the electropherotype of MsReV genome segments; right panel, each genome segment and putative proteins of MsReV.

The filtered tissue homogenates from diseased largemouth bass caused overt cytopathic effects (CPE) in GCF, GCO, CHSE-214 and BF-2 cell lines after three to four days incubation. The temperature range for virus replication extended from 15–25 °C, and the maximum virus infectivity titer was obtained in GCF cell lines with 1 × 10^5.5^ TCID_50_ mL^−1^ at 20 °C. Electron microscopic observations of negatively-stained samples revealed that the virus particles have the typical morphology of aquareoviruses, which have characteristic double-layered capsids and are approximately 70–80 nm in diameter (Figure 1a). This virus is now referred to as largemouth bass *Micropterus salmoides* reovirus (MsReV).

MsReV genomic dsRNAs were extracted from purified virus particles and analyzed by polyacrylamide gel electrophoresis (PAGE). The genomic dsRNAs from two other aquareoviruses, SMReV and GCReV-109, as previously described by our laboratory [24,26], were also prepared and used as molecular weight size markers. The GCReV-109 genome segments have been divided into four size clusters: cluster 1 (S1–S3), cluster 2 (S4–S6), cluster 3 (S7–S9) and cluster 4 (S10 and S11), and the migration pattern of GCReV-109 genome segments described as 3-3-3-2 is typical for grass carp group (freshwater fish) aquareovirus. SMReV genome segments were separated into 10 distinct bands, with segments S1 and S2 comigrating, and the migration pattern of SMReV genome segments described as 3-3-2-2-1, is the other group (saltwater fish) of aquareovirus. Here, the electrophoretic migration pattern of MsReV genomic dsRNA in polyacrylamide gel was shown in Figure 1b (left panel). The genome segments of MsReV were separated into nine distinct bands, with segments S1 and S2, and segments S7 and S8 comigrating, respectively. The migration pattern of MsReV genome segments was clearly identified as 3-3-2-2-1. The characteristics of genome segments and predicted proteins of MsReV were shown in Figure 1b (right panel). The results showed that the electropherotype of MsReV genome segments was similar to that of SMReV, but some changes were apparent in GCReV-109 genome electropherotype.

### 3.2. Organization and Structure of MsReV Genome

The genome segments 1-11 of MsReV were sequenced completely and have been deposited in GenBank under accession numbers KJ740724 to KJ740734. The complete genome sequence of MsReV consists of 24,024 bp divided into 11 segments that range in size from 3947 bp (S1) to 783 bp (S11) (Figure 1b, right panel). The lengths of non-coding regions (NCRs) of MsReV genome segments ranged from 12–28 bp at the 5′ ends, and ranged from 29–162 bp at the 3′ ends. Analysis of the 5′- and 3′-NCRs showed that all of MsReV segments shared conserved nucleotides with 5′-GUUUUA^U^/G/_A_ at their 5′ ends and ^U^/_A_UUCAUC-3′ at their 3′ ends. Moreover, the first and last nucleotides of all segments were complementary (G-C), which are known to be highly conserved within aquareoviruses.

The open reading frame (ORF) analysis revealed that all of MsReV genome segments contained a single ORF, with the exception of S7 segment, which had two partially overlapping ORFs. MsReV was predicted to encode a total of 12 proteins, including seven structural proteins (VP1 to VP7) and five nonstructural proteins (NS87, NS22, NS32, NS38 and NS25) (Figure 1b, right panel). Alignments of the concatenated sequences of seven structural proteins from 13 aquareoviruses revealed that MsReV showed high sequence identities with SMReV (91.2%) and chum salmon reovirus (CHSRV) (85.5%) from hosts in saline environments, but a low level of identity (30.4%–48.5%) with other aquareoviruses from hosts in freshwater environments (Table 1).

### 3.3. Comparison of the S4 Segments and Encoded Nonstructural Proteins of Three Aquareoviruses

**Figure 2:**
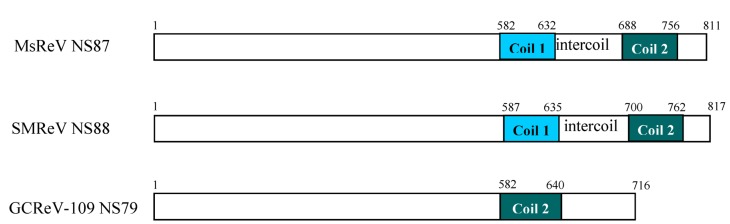
Diagram of predicted coil regions in the nonstructural proteins encoded by S4 segments among MsReV, SMReV and GCReV-109. MsReV NS87 and SMReV NS88 have two coiled coils (Coil 1 and Coil 2) and an intercoil spacing between the two coils, while GCReV-109 NS79 has only one coil (Coil 2). Numbers refer to amino acids residues.

Nucleic acid sequence analysis indicated that the S4 genome segments of MsReV, SMReV and GCReV-109 were predicted to encode three homologous nonstructural proteins (NS87, NS88 and NS79, respectively), which were thought to be involved in the formation of viral inclusion bodies. MsReV NS87 was about 87 kDa, consisting of 811 amino acids, which was a little smaller than SMReV NS88 (88 kDa, 817 aa), but larger than GCReV-109 NS79 (716 aa, 79 kDa). Amino acid sequence alignment showed that MsReV NS87 shared 69.5% identity to SMReV NS88, and only 16.1% identity to GCReV-109 NS79. As predicted by Coils program, MsReV NS87 had two coiled coils (Coil 1, aa 582-632, and Coil 2, aa 688-756) and an intercoil spacing between the two coils, which were also conserved in SMReV NS88 (Figure 2). However, only one coil, corresponding to the regions of Coil 2 in MsReV NS87 and SMReV NS88, was predicted at amino acid positions 582-640 of GCReV-109 NS79 (Figure 2).

### 3.4. Experimental Verification of the Function of NS22 Protein Encoded by MsReV S7

**Figure 3:**
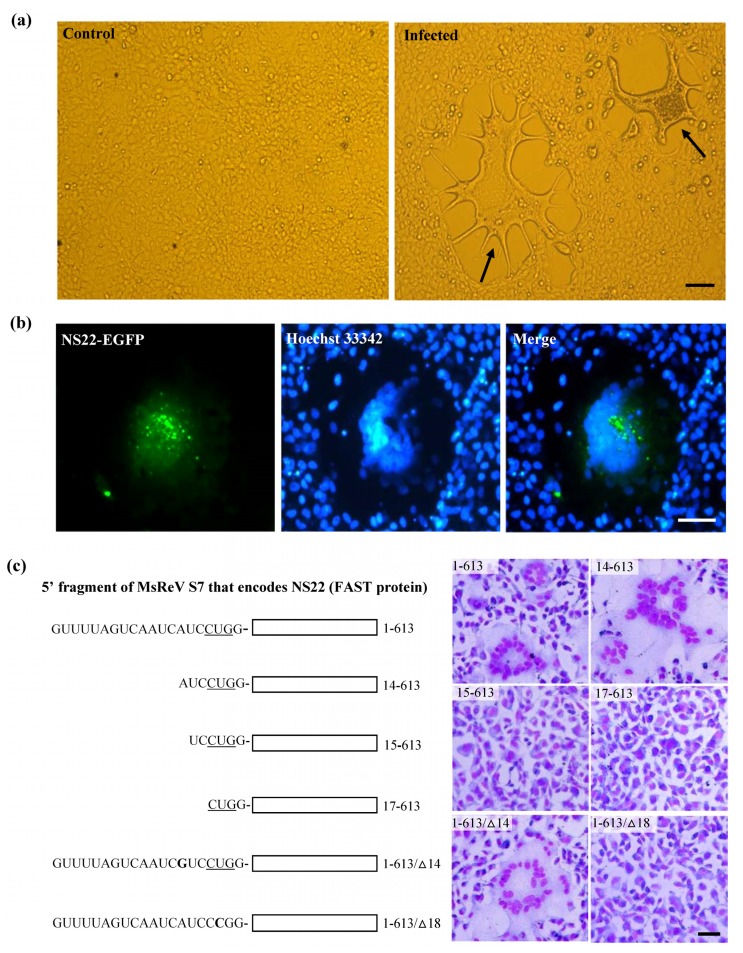

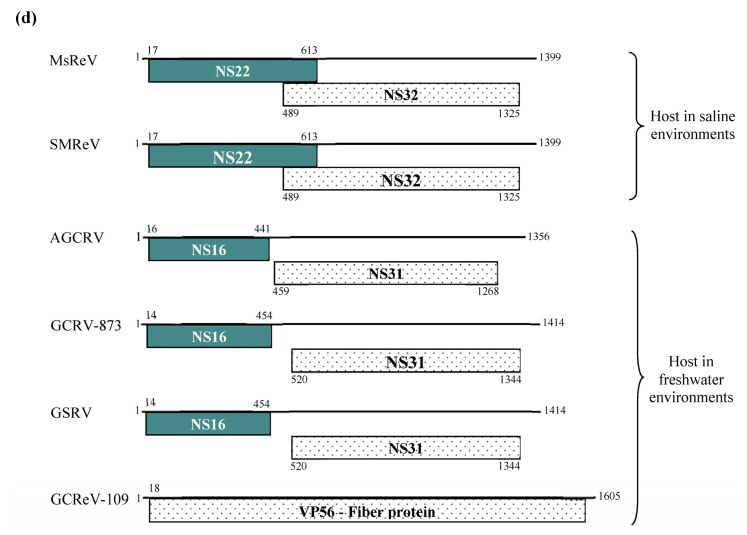
Experimental evidence on the function of MsReV FAST protein NS22. (**a**) Syncytium formation, a characteristic form of cytopathic effect (CPE) induced by MsReV in GCF cell lines at 72 h after infection. Arrows indicate the syncytia. Bar = 200 μm; (**b**) Fluorescence micrographs of GCF cells transfected with plasmid pEGFP-NS22. The expression of NS22-EGFP induced syncytium formation, while NS22-EGFP was distributed in the fused cells. Bar = 100 μm; (**c**) A series of recombinant plasmids containing different regions of S7 (5′ fragment of MsReV S7 that encodes the NS22), including full NA22-coding gene (1-613), deletions (14-613, 15-613, 17-613) and point mutations (1-613/∆14, 1-613/∆18). The putative start site (CUG) is underlined. GCF cells were transfected with the indicated constructs, and stained by Wright-Giemsa staining at 48 h post transfection. Representative images are present at the right. Expression of functional NS22 (1-613, 14-613 and 1-613/∆14) could lead to the production of syncytia, but the non-functional NS22 (15-613, 17-613 and 1-613/∆18) did not induce visible changes. Bar = 100 μm; (**d**) Each S7 genome segment organization and their encoded proteins in the reported aquareoviruses. NS22 ORFs are shown as shaded boxes in MsReV and SMReV, and NS16 ORFs are shown as shaded boxes in AGCRV, GCRV-873, and GSRV. But GCReV-109 lacks the NS22 ORF. Numbers indicate the size of the S7 genome segments and the first and last nucleotides of each ORF.

Initial infection assay showed that GCF cell line was susceptible to MsReV, and the infected cells displayed typical CPE characterized by cell–cell fusion and syncytium formation (Figure 3a). Subsequently, a fusion-associated small transmembrane (FAST) protein NS22 was identified in MsReV S7 segment by sequence analysis. The FAST protein is a viral nonstructural protein, which has membrane-destabilizing activity that may contribute to cell–cell fusion and syncytium formation in virus-infected cells. To confirm the ability of MsReV NS22 to induce cell–cell fusion, we generated series of recombinant plasmids which contain different regions of S7, including the full gene encoding NS22 with enhanced green fluorescent protein (pEGFP-NS22) or without EGFP (1-613), deletions (14-613, 15-613, and 17-613) and point mutations (1-613/∆14 and 1-613/∆18) for protein expression in GCF cells. Expression of the full gene (NS22-EGFP and 1-613), deletion (14-613) and point mutation (1-613/∆14) induced multinucleated syncytia formation in GCF cells (Figure 3b,c), but the deletions (15-613) and (17-613) did not have any noticeable effect on the phenotype in the cells. Furthermore, for detection of NS22 activity, the start codon for NS22 ORF was disrupted by changing a single nucleotide at 18 (^17^C**U**G^19^ to C**C**G). The gene expression that alteration of the translational start site prevents synthesis of MsReV NS22 and syncytium formation in transfected cells were assessed, point mutation (1-613/∆18) could not produce syncytia (Figure 3c). The experiments showed that MsReV NS22 encoded by S7, which is translated from a CUG start codon, indeed, contributed to syncytial cytopathic effect. The S7-coded proteins (e.g., NS22, NS32) of MsReV and other aquareoviruses were depicted and compared. MsReV NS22 showed high similarity in structure and function of SMReV NS22 previously reported by our laboratory [26], and they were clustered in one subgroup (host in saline environments). The small size NS16 proteins were clustered in another subgroup, including American grass carp reovirus (AGCRV), golden shiner reovirus (GSRV) and GCRV-873 (host in freshwater environments), or even lacked the corresponding gene from GCReV-109 (host in freshwater environment) (Figure 3d).

### 3.5. MsReV and SMReV Are Closely Related to Equivalent Genome Segments

**Figure 4:**
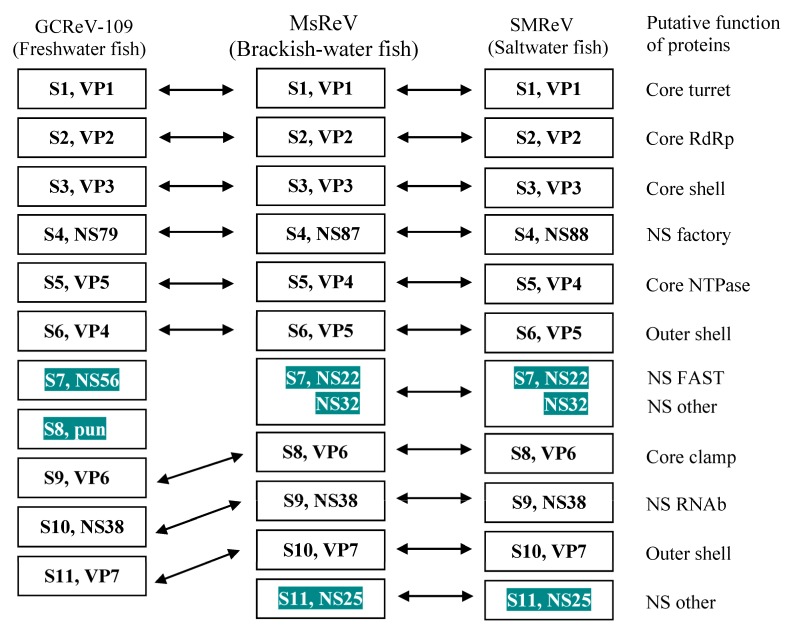
Equivalent genome segments and their encoding proteins among MsReV, SMReV and GCReV-109. Double-headed arrows indicate the equivalent segments and proteins. The shaded boxes show consistency between MsReV and SMReV (S7, NS22 and NS32; S11, NS25), but differences with GCReV-109 (S7, NS56; S8, pun). Pun, protein unknown function.

Functionally equivalent genome segments from the three aquareoviruses, MsReV, SMReV and GCReV-109 were analyzed. The MsReV and SMReV genome segments encode 12 proteins, respectively, which consist of seven structural proteins (VP1 to VP7) and five nonstructural proteins (NS87 or NS88, NS22, NS32, NS38 and NS25). However, GCReV-109 genome segments encode only 11 proteins, which consist of seven structural proteins and four nonstructural proteins (NS79, NS56, pun and NS38). GCReV-109 lacks the genes encoding the nonstructural proteins (NS22 and NS25), and S8 encodes a protein of unknown function (pun) that has no equivalent protein in MsReV and SMReV. These results showed that MsReV was more closely related to SMReV than to GCReV-109 (Figure 4).

### 3.6. MsReV Is More Closely Related to SMReV than to GCRV-109

**Figure 5:**
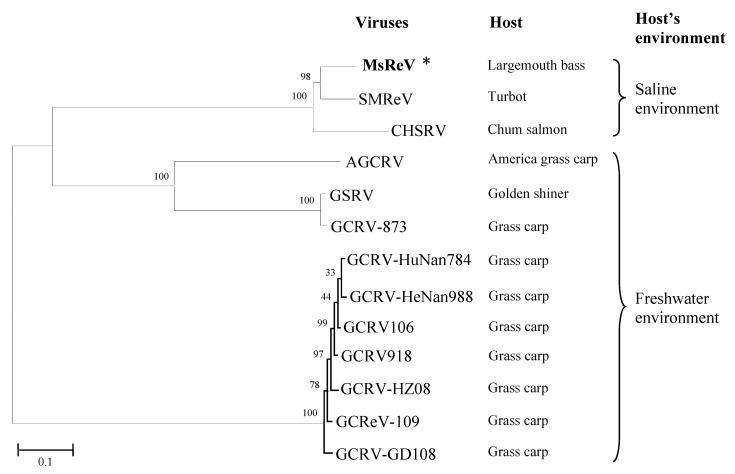
Phylogenetic tree of aquareoviruses based on concatenated sequences of seven structural proteins. Bootstrap values (%) for 1000 replications are shown at branch nodes. The scale bar indicates number of amino acid substitutions per site. Two subgroups, freshwater environments and saline environments (including brackish water and seawater), were determined in aquareoviruses by their host environments.

The analysis above revealed that MsReV and SMReV shared a close relationship through genome anatomy and gene function detection but the correlation between their host environments is poorly resolved. To check the association between aquareoviruses’ phylogenetic background and their host environments, we undertook to further characterize and compare genome segments encoding multiple proteins. Thirteen aquareoviruses were analyzed according to the concatenated sequences of seven structural proteins (VP1 to VP7) (Table 1). The phylogenetic tree showed that the 13 aquareoviruses were divided into two subgroups, one is host in freshwater environments and the other is host in saline environments (Figure 5). MsReV was closely clustered with SMReV in the subgroup of host in saline environments, and GCRV-109 was clustered with AGCRV, GSRV and different GCRV isolates in the subgroup of host in freshwater environments. The evidence that aquareoviruses species closely related also have similar host environments indicated that the high genomic structure similarities between MsReV and SMReV were associated with their hosts in saline environments.

## 4. Discussion

Present research involves whole-genome sequencing and electrophoretic migration pattern of MsReV genome segments, functional identification of proteins, and phylogenetic analysis of concatenated structural protein sequences. These studies revealed marked similarities between the genomic structures of a novel aquareovirus MsReV and SMReV. These aquareoviruses from hosts that had similar environments (e.g., saline environments) were closely related, but alienated from those whose hosts lived in different environments (e.g., freshwater environments). These findings suggest that the aquareoviruses’ genome diversity is associated with their host environments. For example, MsReV, SMReV and CHSRV were isolated from different locations and at different times [26,41], but they were clustered into the same clade in the phylogenetic tree. Thus, the high genomic structure similarities among the three aquareoviruses might have no relation to the space and time of virus isolation, but were related to the physiological environment of their hosts. This is the first report indicating that aquareoviruses’ genomic structures are associated with their host physiological conditions. Broadening our understanding of the genomic diversity of aquareoviruses that exist in different host environments will significantly improve our ability to recognize novel aquareoviruses in the context of aquaculture disease outbreaks.

The fusion-associated small transmembrane (FAST) proteins of the fusogenic reoviruses are the only known examples of membrane fusion proteins encoded by nonenveloped viruses [26,42,43,44]. MsReV S7 encodes the FAST protein NS22 with a CUG start codon, which contributes to syncytial cytopathic effect. The results were confirmed by the experiments of construction and expression of plasmids carrying different regions of S7, and might be further expanded to study not only the response of the MsReV FAST protein function, but also that aquareoviruses were divided into two major branches of host environments according to the FAST protein structure (except for CHSRV). More significantly, investigating the correlation of aquareovirus genetic variants with viral host environments through the FAST proteins could provide the first link between aquareovirus nonstructural protein, gene structure and its host environment, allowing us to establish the relevance and causal relationship of aquareovirus function genes to their host environments.

An increasing number of different reoviruses have been isolated from marine and fresh water in the past years [8,28,34,45]. Emergence of new infectious diseases in lower vertebrates (e.g., fish) or human is not a new phenomenon [46]. Viruses are very genetically diverse and new genotypes, strains and species evolve rapidly [47]. It is important to increase understanding of the virus genomic structure, and several factors are believed to be major reasons for generating genetic variation in RNA viruses, such as mutation [48,49], recombination [50], and reassortment [51,52]. Aquareoviruses’ genomic diversity provides a unique opportunity to examine and explore the mechanisms that are involved in the evolution of multisegmented RNA virus genome and their host environments [4]. Comparative genomic sequencing, functional characterization of two nonstructural proteins combined with functionally equivalent genome segments analysis, and in particular the phylogenetic analysis of the concatenated sequences of seven structural proteins indicated that aquareoviruses’ extensive genomic structural variations between hosts in saline environments and freshwater environments, and host environments similar to aquareoviruses, occur most often between closely related species in natural populations. Although these studies have offered new insights into the host environment’s effect on aquareovirus genomic structure, the mechanisms involved in viral genomic structural variations impacted by host environments need to be further explored.
